# Is assessment good for learning or learning good for assessment? A. Both? B. Neither? C. It depends?

**DOI:** 10.1007/s40037-015-0229-1

**Published:** 2015-10-23

**Authors:** Francois J Cilliers

**Affiliations:** Department of Health Sciences Education, Faculty of Health Sciences, University of Cape Town, Anzio Road, 7925 Observatory, South Africa

The relationship between assessment and learning has long entranced and bewildered teachers and researchers [1, 2]. Anybody with a university degree can relate how they adapted their own learning to assessment and anybody who has taught students will have stories to tell about how students respond to assessment. Yet, no matter how much we believe and profess that ‘assessment drives learning’, are we in a position to harness this supposedly powerful effect?

Researchers have expended a great deal of effort on the relationship between assessment and learning over decades, often in waves of (mostly) descriptive studies comparing the relationship of different methods of assessment with learning. For decades after the description of surface and deep approaches to learning, studies compared whether one assessment method was more or less strongly associated with inventory-based measures of surface and deep approaches to learning than another. Currently, many studies focus on test-enhanced learning.

In this issue, Deng et al. [3] continue a trend of taking the concept of test-enhanced learning into the wilds of everyday practice to explore whether findings from controlled settings translate into studies with greater ecological validity. In keeping with some other literature, their findings suggest that common student learning behaviours that equate to the use of retrieval practice—reviewing multiple choice questions and using flash cards—are indeed associated with positive outcomes on learning in the form of student performance.

From a researcher’s and teacher’s perspective, research on test-enhanced learning contributes to but one segment of a somewhat bewildering array of findings relating assessment and learning. Making sense of these findings has been all the more difficult for the fact that the literature on assessment and learning has been bedevilled by a lack of clarity [4]. When authors write about assessment, what—exactly—do they mean? Formative assessment? Feedback as part of formative assessment? Coursework? Summative assessment? The assessment method being used? And when they write about learning, do they mean student learning behaviours like scheduling time for learning or using flash cards? Do they mean the in-the-brain process of learning i.e., of acquiring and encoding knowledge, skills, dispositions? Or the outcome of that process i.e., later retrieval and performance?

There have been attempts to impose some order on the situation. The concept ‘learning effects of assessment’ provides a useful way to describe and relate an array of effects. The characterization of these effects as pre-assessment effects, post-assessment effects and pure assessment effects [5] helps us to conceptualize what type of learning effects are in play in any given assessment situation. This offers teachers a way to relate different research findings to, say, a mid-term test with feedback. The ways that students adapt their learning to the upcoming test constitute pre-assessment learning effects [4, 6] from the perspective of both learning-as-behaviour and learning-as-process. Retrieval practice [7] during the test and subsequent feedback [8] should both hopefully yield beneficial post-assessment learning effects from the perspective of learning-as-performance.

But what about the students’ perspective? After all, any attempt to utilize research findings in practice will typically occur not in isolation but rather in the context of multiple existing teaching and assessment practices. Any given new assessment practice will become part of an existing, complex web of learning opportunities and assessment practices—often from multiple courses simultaneously—that students are exposed to. How will students react to new practices and why? Will they react at all? Will the relative impact of the new practice be enough to yield an educationally as opposed to a statistically significant result? This may not always be the case [9–11].

Various attempts have been made to model the impact of the learning environment, including assessment, on student learning. I found health behaviour theory to be a useful lens to understand how consequential assessment [12] influences students’ learning in real-life settings. The model I proposed relates a range of aspects of consequential assessment to the pre-assessment quality and regulation of students’ learning. The model incorporates impact appraisal, response appraisal, agency and interpersonal factors, constructs derived from health behaviour theory. It reflects how students responded to varying and changing demands of both the assessment of theory and clinical assessment, relative to other aspects of their academic and personal lives [4, 6, 13]. The model offers one approach to understanding the dynamic interplay between assessment and learning when trying to use assessment purposively to influence learning.

So, is assessment good for learning or learning good for assessment? Why, both, of course! As things stand, there is growing evidence that any assessment practice—formative or summative—will influence learning. There are also increasingly nuanced ways of understanding that oh-so-generic of statements: ‘assessment drives learning’. The question is whether these insights can be harnessed for everyday practice in ways that enhance student learning. The current paucity of reports on the systematic and successful use of assessment to positively influence student learning outside of controlled settings suggests we are not there yet.
